# Complete Genome Sequence of the First Aichi Virus Isolated in Taiwan

**DOI:** 10.1128/genomeA.00107-12

**Published:** 2013-01-24

**Authors:** Jenn-Tzong Chang, Yao-Shen Chen, Bao-Chen Chen, David Chao, Tsung-Hsien Chang

**Affiliations:** a Department of Biological Sciences, National Sun Yat-sen University, Kaohsiung, Taiwan; b Department of Medical Education and Research; c Department of Pediatrics; d Department of Infectious Diseases; e Department of Microbiology, Kaohsiung Veterans General Hospital, Kaohsiung, Taiwan

## Abstract

The first Aichi virus (AiV), kvgh99012632/2010, was identified in Taiwan. The entire genome of the AiV isolate was sequenced and compared to known AiV sequences. Genome alignment revealed that the Taiwan AiV strain shares 96.3% nucleotide and 99% amino acid identities with the German strain D/VI2287/2004.

## GENOME ANNOUNCEMENT

Aichi virus (AiV), one of the members of the family *Picornaviridae*, is a small round-structured nonenveloped virus with a single-stranded and positive-sense RNA genome (1). AiV belongs to the genus *Kobuvirus*, which consists of 3 established species; the other 2 viruses are bovine kobuvirus and porcine kobuvirus (2, 3). AiV was first identified in a stool specimen of a patient with diarrhea due to eating oysters in Japan in 1989 (4). Although AiV resulted mainly in acute gastroenteritis, extraintestinal manifestations, such as respiratory symptoms, were also reported (5, 6). However, the human pathogenicity of AiV is still under study.

In January 2010, the first Taiwanese strain of AiV was identified from a rectal swab of a female neonate with enterovirus-related syndromes in an obstetrics clinic in southern Taiwan (Y.-S. Chen and B.-C. Chen, unpublished report). The strain, named AiV kvgh99012632/2010, was propagated further in Vero cells.

On the basis of the sequences of the AiV strain D/VI2287/2004 available in GenBank (accession no. GQ927711), 11 pairs of oligonucleotide primers were designed to amplify the different regions of the kvgh99012632/2010 genome, including the 5′- and 3′-end fragments. PCR fragments were sequenced in both directions. The complete genome of this virus consists of 8,219 nucleotides (nt), excluding the 3′ poly(A) tail. Flanked by a 679-nt 5′ untranslated region (UTR) and a 244-nt 3′ UTR, the predicted polyprotein is encoded by a 7,296-nt single open reading frame. The genome organization, with 5′ UTR–leader protein-3 structural proteins (VP0, VP3, and VP1)–7 nonstructural proteins (2A, 2B, 2C, 3A, 3B, 3C, and 3D)–3′ UTR, is identical to that of kobuvirus (1). We aligned the genome sequence of kvgh99012632/2010 with 10 complete AiV genomes available in GenBank: AiVs (no. AB040749 and NC_001918), BAY/1/03/DEU (no. AY747174), Chshc7 (no. FJ890523), Goiania/GO/03/01/Brazil (no. DQ028632), D/VI2287/2004 (no. GQ927711), D/VI2244/2004 (no. GQ927712), D/VI2169/2004 (no. GQ927704), D/VI2321/2004 (no. GQ927706), and D/VI2359/2004 (no. GQ927705). AiV kvgh99012632/2010 shared the highest nucleotide sequence homology with D/VI2287/2004 (96.6%), and more than 90% identity with the other isolates. All isolates shared more than 95% amino acid identity; however, kvgh99012632/2010 shared 99% amino acid identity with D/VI2287/2004. Our AiV isolate may belong to genogroup A (7).

AiV kvgh99012632/2010 is the first AiV strain isolated in Taiwan. The virus could be one of the pathogens that causes clinical abnormalities in newborns. The availability of its genome sequence will help facilitate future investigations of the epidemiology and pathogenicity of AiV infection.

### Nucleotide sequence accession number.

The genome sequence of AiV kvgh99012632/2010 has been deposited in GenBank under the accession no. JX564249.
